# From immune checkpoints to therapies: understanding immune checkpoint regulation and the influence of natural products and traditional medicine on immune checkpoint and immunotherapy in lung cancer

**DOI:** 10.3389/fimmu.2024.1340307

**Published:** 2024-02-15

**Authors:** Yibin Zhou, Fenglan Wang, Guangda Li, Jing Xu, Jingjing Zhang, Elizabeth Gullen, Jie Yang, Jing Wang

**Affiliations:** ^1^ Department of Hematology and Oncology, Dongzhimen Hospital, Beijing University of Chinese Medicine, Beijing, China; ^2^ Department of Pharmacology, Yale Medical School, New Haven, CT, United States

**Keywords:** natural product, traditional medicine, lung cancer, immune checkpoint, immunotherapy

## Abstract

Lung cancer is a disease of global concern, and immunotherapy has brought lung cancer therapy to a new era. Besides promising effects in the clinical use of immune checkpoint inhibitors, immune-related adverse events (irAEs) and low response rates are problems unsolved. Natural products and traditional medicine with an immune-modulating nature have the property to influence immune checkpoint expression and can improve immunotherapy’s effect with relatively low toxicity. This review summarizes currently approved immunotherapy and the current mechanisms known to regulate immune checkpoint expression in lung cancer. It lists natural products and traditional medicine capable of influencing immune checkpoints or synergizing with immunotherapy in lung cancer, exploring both their effects and underlying mechanisms. Future research on immune checkpoint modulation and immunotherapy combination applying natural products and traditional medicine will be based on a deeper understanding of their mechanisms regulating immune checkpoints. Continued exploration of natural products and traditional medicine holds the potential to enhance the efficacy and reduce the adverse reactions of immunotherapy.

## 1 Introduction

Lung cancer is the leading cause of cancer death and ranks the second for the incidence rate worldwide, over 45% of lung cancer patients are diagnosed at late stage (1, 2). Chemotherapy and targeted therapy are widely used in advanced lung cancer depending on the status of actionable driver mutations (3). Besides directly killing tumor cells, clinicians are now concentrating on the tumor microenvironment. It is reported that immunosuppressive environment is related to poor treatment results (4). The tumor can evade immune surveillance in this environment and easily metastasize (5). Immune checkpoints are observed to be associated with immunosuppressive environment. Programmed cell death 1 (PD-1) and programmed death-ligand 1 (PD-L1), the most studied immune checkpoints, play essential roles in tumor progression (6, 7). Other immune checkpoints like IDO (Indoleamine-2,3-dioxygenase) and B7-H3 can also inhibit the immune reaction and promote lung cancer progression (8–10). Immune checkpoint inhibitors (ICIs) are now widely used for lung cancer treatment and have shown promising therapeutic activities (11, 12).

Nevertheless, only a few patients can benefit from this therapy, and the expression level of immune checkpoints such as PD-L1 is positively correlated to the effect of ICIs (13). Natural products are promising candidates to promote ICIs efficacy by upregulating immune checkpoints expression. On the other hand, ICIs directly blockade the interaction between immune checkpoints, while natural products can interrupt checkpoints interaction by inhibiting their expression via different mechanisms (14–16). Therefore, understanding the regulatory mechanisms of immune checkpoints in lung cancer and mechanisms of how natural products modulate immune checkpoint expression are extremely significant for exploring novel approaches to inhibit immune checkpoint expression and develop new drugs based on natural products. In this study, we would like to summarize currently known immune checkpoint regulation mechanisms in lung cancer and introduce the effect of natural products and traditional medicine that can influence immune checkpoints or promote immunotherapy in lung cancer.

## 2 Immunotherapy in lung cancer

Immune check point inhibitors pembrolizumab, atezolizumab, or cemiplimab can be used as first-line single-agent for metastasized non-small cell lung cancer (NSCLC) patients with PD-L1 expression level ≥50% and negative driver gene mutation that have recommended first-line targeted therapy according to the NCCN guideline (17). Furthermore, NCCN also recommends that metastatic NSCLC patients with PD-L1 levels of 1% to 49% and negative driver gene mutation to use pembrolizumab alone as a first-line therapy. Meanwhile, immune checkpoint inhibitors are not recommended as single-agents in small cell lung cancer (SCLC).

Besides being used as a single agent in advanced NSCLC, immune checkpoint inhibitors can combine with chemotherapy before or after surgery. CheckMate 816 is a phase 3 randomized trial that discovered neoadjuvant therapy with nivolumab plus platinum-doublet chemotherapy has a longer event-free survival time and higher pathologic complete response rate, major pathologic response rate, and overall response rate versus chemotherapy alone (18). Thus NCCN guidelines recommends nivolumab plus platinum-doublet chemotherapy as neoadjuvant therapy in resectable (tumors ≥4 cm or node positive) NSCLC (17). NCCN guidelines also recommends atezolizumab as an adjuvant therapy for patients who have previously received adjuvant chemotherapy with completely resected stage IIB to IIIA or high-risk stage IIA NSCLC and PD-L1 ≥1%, according to clinical trial results from another phase 3 randomized trial-IMpower010 which compared adjuvant therapy with atezolizumab versus best supportive care in patients with resected early-stage NSCLC (12). For extensive-stage SCLC, atezolizumab plus carboplatin plus etoposide followed by maintenance atezolizumab, or durvalumab plus etoposide plus (carboplatin or cisplatin) followed by maintenance durvalumab as first-line therapy based on the clinical trial data from two phase 3 randomized trials: IMpower133 or CASPIAN (11, 19–21). In SCLC, the NCCN guidelines recommend carboplatin plus etoposide plus atezolizumab as the first-line systemic therapy, followed by maintenance atezolizumab for patients with extensive-stage SCLC, based on the clinical outcomes of the IMpower133 trial (21–23). According to the results from the CASPIAN trial, the NCCN panel recommends durvalumab plus etoposide plus (carboplatin or cisplatin) as a first-line systemic therapy option, followed by maintenance durvalumab for patients with extensive-stage SCLC (20, 22) (11, 24, 25).

Although immunotherapy brings clinical benefits and gets involved in first-line therapy, a large number of patients do not respond to immune checkpoint inhibitors (26, 27). And for immune checkpoint inhibitors used in adjuvant and neoadjuvant therapy, there lacks a clearly defined best target population, which needs further exploration (28). Immune-related adverse events (irAEs) also need further research, the incidence of irAEs across agents and trials ranges from 15% to 90%, and approximately 0.5% to 13% of them happened severe irAEs which need immunosuppression or treatment discontinuation in monotherapy (29, 30). In brieg summary, there are still a lot of unsolved questions in immunotherapy requiring further exploration to bring patients better outcomes and fewer adverse events.

## 3 Immune checkpoints regulation in lung cancer

**Figure 1:**
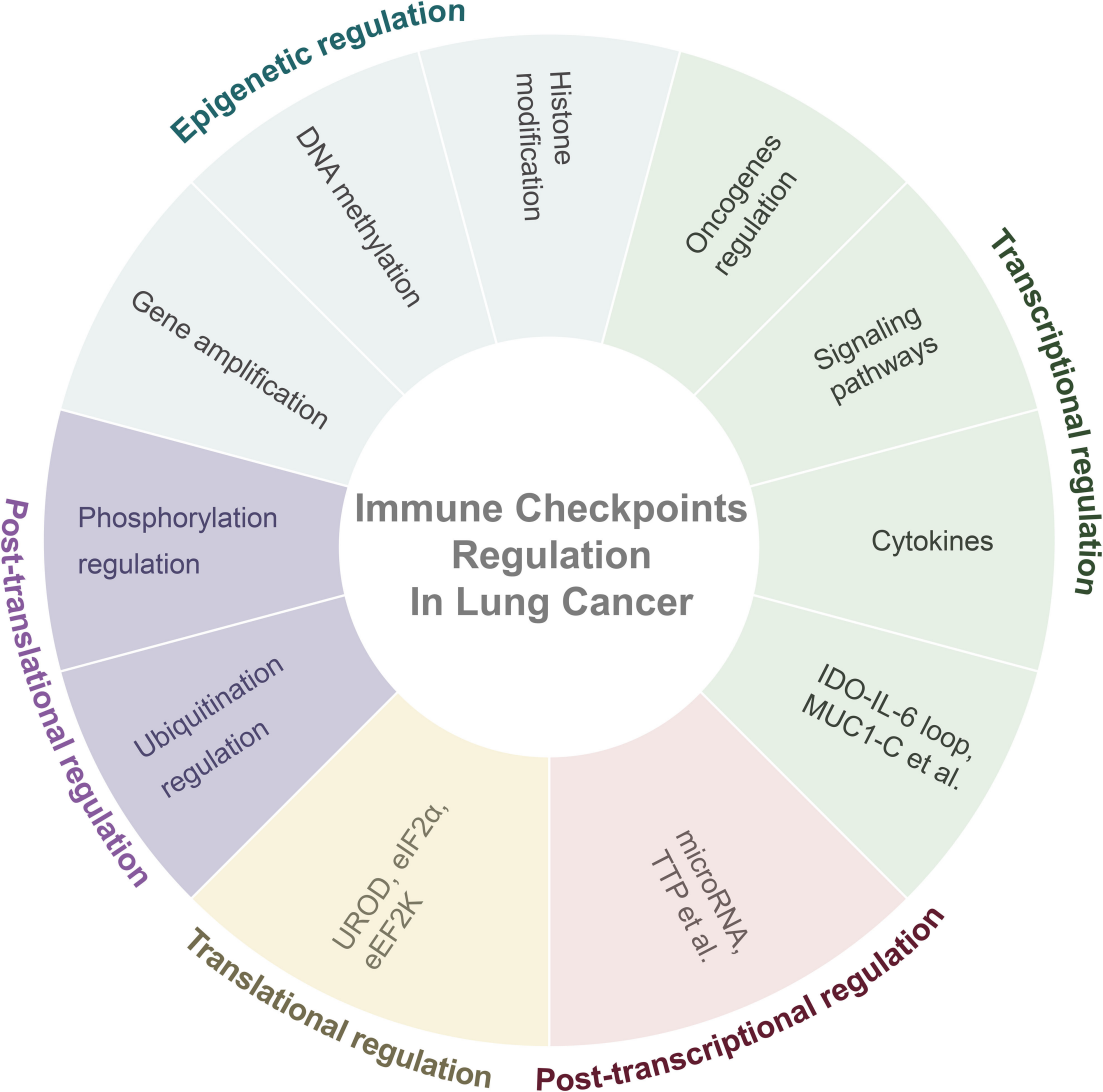
Overall regulatory mechanisms of immune checkpoints in lung cancer. Abbreviation: IDO= Indoleamine-2,3-dioxygenase; IL-6= Interleukin-6; MUC1-C= Mucin 1-C; TTP= AU-rich element-binding protein tristetraprolin; UROD= Uroporphyrinogen Decarboxylase; eEF2K= Eukaryotic Elongation Factor 2 Kinase.

Most study of immune checkpoint regulation in lung cancer has focused on the regulation of PD-L1. Therefore, in this section, the regulatory mechanisms of PD-L1 are primarily discussed (**Figure 1**).

### 3.1 PD-L1 regulation

#### 3.1.1 Epigenetic regulation

Epigenetic regulation can alter chromatin structure and control the expression of PD-L1 by modulating the recognition and binding between transcription factors and DNA elements instead of altering the DNA sequence (31), for instance, epigenetic regulation of lung cancer PD-L1 involves gene amplification, DNA methylation, and histone modifications.

##### 3.1.1.1 Gene amplification

In NSCLC, around 1.9% of patients have PD-L1 gene amplification and approximately 8.2% of them exhibit PD-L1 gene amplification at 9p24.1 which is associated with the JAK-STAT pathway and concurrent amplification of the JAK2 gene (32–34).

##### 3.1.1.2 DNA methylation

DNA methylation involves the covalent modification of cytosine at the 5-position of nucleotides (35), regulates gene expression by controlling chromatin structure, DNA stability, and conformation (36). Study showed that TGF-β1 can inhibit the activation of DNMT1 (DNA methyltransferase-1), thereby demethylating the CD274 (PD-L1) promoter to upregulate PD-L1 expression (37). However, studies suggest that methylation negatively correlates with PD-L1 expression in NSCLC biopsies, yet the correlation is relatively weak, the role of methylation in PD-L1 expression remains contentious and the potential benefits of demethylation therapy combined with immunotherapy remain debatable (38).

##### 3.1.1.3 Histone modification

Histone methylation plays a role in regulating the expression of PD-L1. Combined with histone methyltransferase, EZH2 (Enhancer of Zeste homolog2) can suppress PD-L1 expression by increasing trimethylation of histone H3K27 at the promoter, which is a regulatory mechanism that depends on HIF-1α in lung cancer (39).

Histone acetylation also plays a role in the regulation of PD-L1 expression. In drug-resistant A549/CDDP cell line, the downregulation of COP1 (E3 ligase constitutive photomorphogenesis protein 1) promotes the activation of the JNK/c-Jun pathway, subsequently inhibiting the expression of histone deacetylase 3 (HDAC3). This ultimately enhances the acetylation of histone H3 at the CD274 promoter resulting in the upregulation of PD-L1 expression (40). Also, a positive correlation has been observed between elevated PD-L1 expression in cisplatin-resistant NSCLC patients and c-Jun expression while a negative correlation exists with HDAC3 (40).

However, another study suggests that the application of HDAC inhibitors can downregulate PD-L1 expression levels in afatinib-resistant NSCLC patients (41). Another research observed a positive correlation between HDAC10 and PD-L1 expression levels in NSCLC patients through immunohistochemistry (42). These findings contradict results mentioned above, indicating the need for a further understanding of the role histone acetylation playing in PD-L1 regulation.

#### 3.1.2 Transcriptional regulation

##### 3.1.2.1 Oncogenes regulation

###### 3.1.2.1.1 KRAS

KRAS mutations have been found to upregulate PD-L1 expression through the MAPK pathway, in which AP-1 family plays a vital role. Chen et al. found that the upregulation of PD-L1 in KRAS mutation lung adenocarcinoma is correlated with p-ERK regardless of p-AKT (43). Sumimoto et al. further discovered that KRAS mutations enhance PD-L1 transcription through the MAPK pathway by binding the downstream AP-1 component cJUN to the CD274 enhancer (44). KRAS mutations were identified as an independent factor inducing PD-L1 expression in the study of premalignant human bronchial epithelial cells, regulates PD-L1 expression through the MEK-ERK pathway and part of the MAPK pathway, with downstream involvement of the AP-1 family member FRA1 (FOS-related antigen 1) (45).

###### 3.1.2.1.2 EML4-ALK

The EML4 (Echinoderm microtubule-associated protein like-4) - ALK (anaplastic lymphoma kinase) fusion has been detected in approximately 4-8.1% of NSCLC patients (46, 47). A study discovered lung adenocarcinoma patients with EML4-ALK fusion have higher levels of PD-L1 on tumor cells than patients without EML4-ALK fusion. The expression of PD-L1 of patients with the EML4-ALK fusion is positively correlated with p-ERK, p-STAT3, and p-AKT (48). Hong et al.’s research indicates that EML4-ALK fusion upregulates PD-L1 expression through the p-ERK1/2 and p-AKT signaling pathways, but it is not associated with the JAK3/STAT3 pathway (49). A study of lung adenocarcinoma demonstrated that EML4-ALK fusion can upregulate p-STAT3 levels, then p-STAT3 upregulates PD-L1 expression by binding to the CD274 promoter. Additionally, EML4-ALK fusion can upregulate HIF-1α under hypoxic conditions, and inhibit HIF-1α ubiquitination and degradation. Then HIF-1α binds to the CD274 promoter to upregulate PD-L1 expression. The authors also emphasized the synergistic role of HIF-1α and STAT3 in promoting PD-L1 expression under hypoxic conditions (50). MAPK, JAK-STAT, and PI3K-AKT pathways involved in EML4-ALK regulated PD-L1 expression, and HIF-1α effective at specific conditions.

###### 3.1.2.1.3 EGFR

Akbay et al. initially mentioned a positive correlation between EGFR mutations and PD-L1 expression, however, their study did not further focus on EGFR mutations’ mechanisms regulating PD-L1 expression until subsequent research explored that (51). Studies suggested that EGFR mutations can upregulate PD-L1 expression through the AKT-STAT3 and p-ERK1/2/p-c-Jun pathways (52, 53). Okita et al. found that the baseline expression of PD-L1 in NSCLC was positively correlated, while EGFR levels negatively correlated with HER2 levels. However, EGF-induced PD-L1 expression was merely associated with EGFR levels. Both baseline and EGF-induced PD-L1 expression were partially associated with the PI3K/AKT and JAK/STAT pathways (54). In EGF-stimulated EGFR mutant NSCLC, EGF induced IL-6 secretion, and IL-6, subsequently induced PD-L1 expression through the JAK/STAT3 pathway (55). Guo et al.’s research suggested that EGFR mutations regulate PD-L1 expression through multiple pathways with interconnections among them, including PI3K-AKT-mTOR-HIF-1α, NF-κB, and the MAPK pathway (56). In lung adenocarcinoma with EGFR mutations, the EMT-related receptor tyrosine kinase AXL was positively correlated with PD-L1 expression. Inhibition of AXL kinase activity could downregulate PD-L1 mRNA (57, 58). Other studies put forward that in EGFR tyrosine kinase inhibitors (TKIs) resistant cells, PD-L1 levels were positively correlated with E-cadherin and EGFR phosphorylation levels, yet the exact mechanism remains unrevealed (59, 60). To summarize, EGFR mutation regulates PD-L1 mainly through JAK-STAT, PI3K-AKT, MAPK, and NF-κB pathways that interconnect with each other. However, negative correlation between PD-L1 expression and EGFR mutation levels are also proposed, so the regulation of PD-L1 by EGFR mutations requires further research (61).

PD-L1 expression in EGFR mutant lung cancer cells is generally higher than that in wild-type (62). However, research found that PD-L1 expression was associated with the STAT3, AKT, and ERK pathways in wild-type EGFR NSCLC, and ubiquitin ligases Cbl-b and c-Cbl could downregulate PD-L1 expression by inhibiting phosphorylation of STAT3/AKT/ERK pathways (63). The TUSC2 (also known as FUS1) gene can downregulate the activation of multiple tyrosine kinases, including EGFR (64, 65). Cao et al. found that TUSC2 in NSCLC could inhibit mTOR, thereby downregulating PD-L1 expression, and Dai et al. suggested that TUSC2 restoration in wild-type EGFR could downregulate PD-L1 expression through the mTOR pathway (66, 67). In wild-type EGFR cells, tumor cell-intrinsic CTLA4 could upregulate PD-L1 expression through the MEK-ERK pathway downstream of EGFR (68). Stutvoet et al. studied on non-EGFR mutant lung adenocarcinoma, found that EGF could induce IL-6 secretion in cells. IL-6, eventually induced PD-L1 expression through the STAT1 pathway. The authors also suggested that the MAPK pathway plays an essential part in regulating PD-L1 expression, as it can upregulate PD-L1 mRNA expression and increase the stability of PD-L1 mRNA (69). Generally speaking, PI3K-AKT, JAK-STAT, and MAPK are the pathways that participate in the regulation of PD-L1 in wild-type EGFR lung cancer.

Some studies have shown that EGFR TKIs can downregulate PD-L1 expression levels. EGFR combined with gefitinib can lower the expression of PD-L1 in EGFR mutant NSCLC through the NF-κB pathway (62, 70). However, Okita et al.’s research indicated that gefitinib cannot downregulate baseline PD-L1 expression which caused by EGFR mutations, but can inhibit EGF-induced upregulation of PD-L1. EGFR TKIs’ mechanism of regulating PD-L1 still remains unknown (54).

Although most studies suggested a positive correlation between EGFR mutations and PD-L1 expression, some studies indicated that EGFR mutant patients have a lower response to PD-1/PD-L1 treatment (71–74). Anti-PD-L1 immunotherapy and EGFR TKI treatment showed no synergistic effect but increasing toxicity in clinical studies, which suggests more exploration of the regulation of PD-L1 by EGFR in clinical applications (75, 76).

###### 3.1.2.1.4 MYC

The MYC oncogene is overexpressed in 41% of NSCLC and is associated with the loss of cell differentiation (77). Studies have found a positive correlation between MYC expression levels and PD-L1 levels. MYC can directly bind to the PD-L1 gene promoter, then promote PD-L1 expression (78–80).

###### 3.1.2.1.5 PTEN

PTEN (phosphatase and tensin homolog) is a gene that functions as a tumor suppressor by inhibiting the PI3K-AKT pathway. It is one of the most common mutated tumor suppressor genes (81, 82). In lung squamous cell carcinoma, approximately 15% of cases exhibit PTEN mutations (83). Studies have shown that PTEN loss in lung cancer is associated with high PD-L1 expression (84). The activation of the PI3K-AKT pathway, regulated by PTEN, is often linked to the upregulation of PD-L1 (4, 85). PTEN knockout significantly upregulates the expression of PI3K-AKT pathway (81). Therefore, current research suggests that the functional impairment of PTEN primarily upregulates PD-L1 expression through the PI3K-AKT pathway.

##### 3.1.2.2 Signaling pathways

###### 3.1.2.2.1 MAPK pathway

The MAPK pathway regulates PD-L1 expression in KRAS mutation NSCLC and EGFR mutation mentioned previously. Studies by Della Corte and Demuth have shown that the MAPK pathway can control PD-L1 expression at the transcriptional level by regulating PD-L1 mRNA levels (86, 87). It is found that MAPK can stimulate PD-L1 transcription through NF-κB as it can bind to the CD274 promoter to promote PD-L1 transcription, which suggests complex regulatory interactions among different pathways in PD-L1 regulation (86). The authors’ research further indicates that inhibiting key factors of the MAPK pathway, such as MEK, can increase the immunogenicity of tumor cells leading to the upregulation of MHC-I (major histocompatibility complex class-I) expression and increased mRNA levels of IFN-γ, IL-6, IL-1B, and TNF-α. Conversely, it downregulates the expression of other immune checkpoint molecules like CTLA-4, TIM-3, and LAG-3, thereby promoting various immune responses (86).

In addition to EGFR and KRAS mutations, MET (mesenchymal-epithelial transition factor) amplification positively correlates with PD-L1 expression. Inhibiting MET expression can downregulate PD-L1 levels (88, 89). MET also regulates PD-L1 expression through the MAPK pathway (87). Nevertheless, MET primarily regulates PD-L1 via the MAPK and PI3K/AKT pathways rather than the NF-κB pathway in EGFR-TKI-resistant NSCLC cells and whether NF-κB is involved in the MAPK pathway’s regulation of PD-L1 requires further investigation (90).

###### 3.1.2.2.2 Hippo pathway

The Hippo pathway regulates tumor immunity highly associated with cancer (91). Several studies have indicated activation of the Hippo pathway proteins YAP (Yes-associated protein) and TAZ (WW domain-containing transcription regulator 1) can upregulate PD-L1 expression in NSCLC. Further research has shown that YAP and TAZ can form complexes with TEAD family proteins, bind to the CD274 promoter or enhancer, thus enhance PD-L1 expression (92–95). Other upstream factors like PKA (protein kinase A) and LATS (large tumor suppressor) also inhibit YAP and TAZ. In this regard, PD-L1 expression can be promoted by upregulating TAZ/YAP (92, 95).

###### 3.1.2.2.3 ADORA1-ATF3 pathway

The ADORA1-ATF3 pathway also modulates the transcriptional regulation of PD-L1. Research by Liu et al. indicates that ADORA1 (adenosine A1 receptor) inhibition leads to increase in ATF3 (cAMP-dependent transcription factor 3) levels, which binds to the CD274 promoter and upregulates PD-L1 expression (96).

##### 3.1.2.3 Cytokines

###### 3.1.2.3.1 IFN-γ

The cytokine IFN-γ is considered as a primary inducer of PD-L1 expression in various tumors (97, 98). Several studies have suggested that IFN-γ promotes the expression of IRF-1 (interferon regulatory factor-1) by activating the JAK-STAT pathway in NSCLC. IRF-1 can bind to the CD274 promoter and facilitate PD-L1 transcription (99). Meanwhile, IRF-1 also plays a crucial role in both constitutive and IFN-γ-induced PD-L1 expression (100).

Lv et al. found that the JAK-STAT pathway activated by IFN-γ can promote the binding of TET1 (ten-eleven translocation methylcytosine dioxygenase 1) to IRF-1, regulating IRF-1 demethylation and thereby promoting PD-L1 expression (101). Lai et al. demonstrated a negative correlation between the methylation levels of IRF1/7 and PD-L1 expression and Decitabine demethylates IRF1/7 leading to the restoration of PD-L1 expression levels (102). In contrast to IRF-1, IRF-2 competitively binds to the CD274 promoter, inhibiting IRF-1’s ability to promote PD-L1 expression (103).

Gao et al. discovered that IFN-γ affects downstream IRF-1 to regulate PD-L1 expression through the JAK2-STAT1 pathway, and participates in regulation through the PI3K-AKT pathway. Further research indicated that the PI3K-AKT pathway also acts through STAT1 to control PD-L1 expression in IFN-γ-induced PD-L1 expression, emphasizing the primary role of the JAK-STAT pathway and it suggested that STAT3 does not participate in IFN-γ-mediated PD-L1 regulation (104). It is mentioned before in EGFR also demonstrated that IFN-γ can induce PD-L1 expression through STAT1, and inhibition of the EGFR pathway could partially affect IFN-γ-mediated PD-L1 expression (69). However, another research suggested that IFN-γ upregulates PD-L1 expression in lung adenocarcinoma cells through the PI3K/AKT and JAK/STAT3 pathways, which need for further research support to prove the potential of STAT3 in IFN-γ induced PD-L1 expression (105).

Besides IFN-γ, Morimoto et al. demonstrated that type I interferon IFN-β increases STAT1 mRNA levels and ultimately upregulates PD-L1 expression via IRF9 through the JAK1/2-STAT1 pathway and independently of the mTOR pathway (106).

###### 3.1.2.3.2 HIF-1α

Hypoxia-inducible factor-1α (HIF-1α) is positively correlated with PD-L1 mRNA and protein expression in NSCLC (56, 107, 108). In myeloid-derived suppressor cells (MDSCs) of LLC (Lewis Lung Carcinoma) mice, HIF-1α can directly bind to the hypoxia-response element (HRE) in the CD274 promoter, promoting PD-L1 transcription (109). As mentioned earlier, it has also been observed that HIF-1α can bind to the CD274 promoter to upregulate PD-L1 expression in EML4-ALK fusion NSCLC (50).

###### 3.1.2.3.3 TGF-β

TGF-β is known to promote tumor epithelial-mesenchymal transition (EMT) and inhibit anti-tumor immunity (110). It can upregulate the expression of DC cell immune checkpoints PD-L1 in an *in vitro* lung cancer microenvironment model (111). Researchers found that TGF-β and TGF-β1 can increase PD-L1 expression in NSCLC cells (112, 113).

David et al. further investigated how TGF-β1 upregulates PD-L1 expression. Their research indicates that TGF-β1 upregulates PD-L1 expression by enhancing PD-L1 transcription levels rather than increasing PD-L1 mRNA stability. This upregulation process might be dependent on Smad2. They found that the transcriptional start site of CD274 has Smad-binding elements, suggesting that Smad2 might upregulate PD-L1 expression by binding to the CD274 promoter, and further research is needed to validate these results (114). The study also suggests that in A549 cells, TGF-β1 may partially upregulate PD-L1 expression through the PI3K pathway.

Interestingly, the research also indicates that TGF-β1 can regulate microRNAs associated with PD-L1 mRNA regulation despite experimental results suggesting that TGF-β1 cannot increase the stability of PD-L1 mRNA (114). More research is needed to understand how TGF-β regulates PD-L1 expression. Conversely, PD-L1 can activate the TGF-β/Smad pathway and participate in primary resistance to EGFR-TKIs in EGFR mutant NSCLC cells (115).

Moreover, studies have shown that TGF-β can impede the efficacy of anti-PD-1/PD-L1 treatments, and the immunosuppressive effects of TGF-β and PD-L1 are independent and complementary (116, 117). Combining anti-TGF-β with anti-PD-1/PD-L1 therapies enhances the therapeutic effectiveness and overcomes treatment resistance (118–121). Therefore, the development of anti-TGF-β/PD-L1 bispecific antibodies, such as YM101, BiTP, and M7824, represents a valuable direction in anti-tumor research (122–126).

###### 3.1.2.3.4 Other cytokines

Other cytokines, such as IL-27, have been shown to upregulate PD-L1 mRNA and cell surface protein levels in A549 cells (127). Nevertheless, not all cytokines positively regulate PD-L1 expression. For instance, research by Schalper et al. demonstrated that IL-10 does not impact PD-L1 expression, while Gao et al.’s study found that IL-10 can inhibit IFN-γ-induced STAT1 phosphorylation in lung adenocarcinoma, thereby suppressing IFN-γ-induced upregulation of PD-L1 (128, 129).

Other mechanisms in transcriptional regulation include MUC1, a frequently overexpressed transmembrane glycoprotein in NSCLC. Its subunit, MUC1-C, can form a complex with NF-κB’s p65 subunit and bind to the CD274 promoter, thereby enhancing PD-L1 expression (130).

#### 3.1.3 Post-transcriptional regulation

Post-transcriptional regulation of PD-L1 is primarily achieved through binding to the 3’ untranslated region (3’ UTR) of PD-L1 mRNA. The factors involved in this regulation are primarily microRNAs (miRNAs) and a subset of other regulatory factors.

MicroRNAs (miRNAs) generally exert their inhibitory effects on target genes by binding to the 3’ UTR of mRNA, thereby promoting their degradation or inhibiting their transcription. Experimental evidence has demonstrated that microRNAs miR-34, miR-140, let-7, miR-200, and miR-200a-3p can bind to the 3’ UTR of PD-L1 mRNA, thereby downregulating the expression of PD-L1. miR-155-5p theoretically possesses binding sites in the 3’ UTR of PD-L1 mRNA but lacks experimental validation (131–137).

Other factors can regulate the expression of PD-L1 by modulating the levels of these miRNAs. For instance, wild-type p53 can bind to the promoter of miR-34, upregulating its expression (136). Long non-coding RNAs (lncRNAs) often act as molecular sponges, directly inhibiting miRNA function: Circ-CPA4 inhibits let-7 (132), MALAT1 inhibits miR-200a-3p (133), lncRNA LINC01140 can inhibit miR-377-3p and miR-155-5p, which are predicted to bind to the 3’ UTR of PD-L1 mRNA (138). MiR-142 has potential binding sites in PD-L1 mRNA and can downregulate PD-L1 expression, while lncRNA FGD5-AS1 acts as a molecular sponge directly interacting with miR-142 (139). One of Circular RNA (circRNA) named hsa_circRNA_002178 can also sequester miR-34 (140). Additionally, the non-RNA factor ZEB1 (zinc-finger E-box-binding homeobox 1) can relieve the inhibitory effect of miR-200 on PD-L1 expression (137).

Apart from miRNAs, other factors can directly act on PD-L1 mRNA. One such factor is TTP (AU-rich element-binding protein tristetraprolin), which can bind to the 3’ UTR of PD-L1 mRNA and inhibit PD-L1 expression (141). In addition, the AU-rich element-binding protein HuR, regulated by the Ang II (Angiotensin II)/AGTR1 pathway, can stabilize PD-L1 mRNA by binding to its 3’ UTR, thereby upregulating PD-L1 expression (142). Furthermore, research indicates that variant single nucleotide polymorphisms within the binding sites in the 3’ UTR of PD-L1 mRNA can disrupt this inhibitory effect (143).

Interestingly, miRNAs can not only directly regulate PD-L1 expression at the post-transcriptional level but can also indirectly influence PD-L1 by modulating related pathways. For instance, miR-135 can promote the phosphorylation of kinases in the JAK/STAT pathway, leading to an upregulation of PD-L1 expression (144). MiR-3127-5p has been found to increase PD-L1 expression by promoting STAT3 phosphorylation (145). MiR-181a and miR-940, by inhibiting the ubiquitin ligase Cbl-b and c-Cbl, activate the STAT3/AKT/ERK pathway, resulting in elevated PD-L1 levels (63). Additionally, miR-197 downregulates PD-L1 expression by inhibiting the CKS1B/STAT3 pathway (146).

In addition, the long non-coding RNA (lncRNA) NKX2-1-AS1 can inhibit the transcription of CD274 (PD-L1) by preventing the binding of NKX2-1 protein to the CD274 promoter (147). LncRNA SNHG12 enhances the stability and expression of PD-L1 and USP8 mRNA by binding to HuR. Simultaneously, USP8, through mediating deubiquitination, stabilizes PD-L1 protein (148).

#### 3.1.4 Translational regulation

Currently, there is limited research on the translational regulation of NSCLC PD-L1. Suresh et al.’s study suggests that the deficiency of Uroporphyrinogen Decarboxylase (UROD) leads to a shortage of heme, which in turn activates an eIF2α kinase called Heme-Regulated Inhibitor (HRI). Then, phosphorylated eIF2α triggers a cascade of other kinases, inducing the integrated stress response (ISR) and global translation initiation inhibition. Under this stress, PD-L1 mRNA is preferentially translated. Moreover, eIF2α promotes the bypass of inhibitory upstream Open Reading Frames (uORFs), thereby facilitating PD-L1 translation (149). In another study, Wu et al. demonstrated that Eukaryotic Elongation Factor 2 Kinase (eEF2K) enhances the association between PD-L1 mRNA and active polysomes, leading to an upregulation of PD-L1 levels (150).

#### 3.1.5 Post-translational regulation

Post-translational regulation of PD-L1 refers to the modulation of the PD-L1 protein. It primarily involves processes such as ubiquitination, phosphorylation, glycosylation, palmitoylation, acetylation, and others, all contributing to regulating PD-L1 at the protein level.

##### 3.1.5.1 Ubiquitination regulation

Ubiquitination regulation encompasses both deubiquitination and ubiquitination of PD-L1. Deubiquitinase USP22 deubiquitinates PD-L1, preventing its proteasomal degradation (151). Furthermore, USP22 stabilizes CSN5, another deubiquitinase, through its deubiquitination activity. CSN5 has been shown in other tumors to stabilize PD-L1 protein through deubiquitination (152). And Wang et al. suggested that USP22 and CSN5 mutually enhance each other’s stabilizing effect on PD-L1 (151). Zhu et al.’s research revealed that deubiquitinases can upregulate PD-L1 protein levels in A549 cells. Their subsequent studies in other non-NSCLC cell lines found that deubiquitinase OTUB1 deubiquitinates PD-L1 by cleaving K48-linked poly-ubiquitin chains, stabilizing PD-L1. This study confirmed that OTUB1 can prevent the endoplasmic reticulum-associated degradation of PD-L1 (153).

However, not all deubiquitinases act to stabilize PD-L1. Xiong et al.’s research indicates that deubiquitinase USP8 downregulates PD-L1 protein levels by targeting K63-linked deubiquitination instead of K48-linked deubiquitination. More specifically, K63-linked deubiquitination antagonizes K48-linked deubiquitination, and deubiquitination of K63 leads to an increase in K48 ubiquitination. Therefore, USP8’s action is opposite to that of OTUB1, which deubiquitates PD-L1 at K48, promoting the degradation of PD-L1 protein (154). Nevertheless, USP8 mentioned above in lncRNA research suggests it can stabilize PD-L1 protein, so whether USP8 promotes or inhibits PD-L1 expression remains further explored (148).

Research on PD-L1 ubiquitination regulation brings up new points. The E3 ubiquitin ligase MARCH8 can interact with the N-terminal region of PD-L1 and ubiquitinate it, promoting the proteasomal degradation of PD-L1 (155). In other tumor types, members of the CMTM protein family have been shown to inhibit the ubiquitination of PD-L1 (156). In NSCLC-related research, CMTM6 has been found to co-localize with PD-L1, preventing PD-L1 from becoming a target of lysosome-mediated degradation (157). On the other hand, E3 ubiquitin ligase TRAF6 promotes K63 ubiquitination to stabilize PD-L1 (154).

##### 3.1.5.2 Phosphorylation regulation

Phosphorylation and ubiquitination of the PD-L1 protein are closely interconnected. Wu et al. found that the EGFR inhibitor ES-072 can downregulate PD-L1 levels, and this process mainly depends on the ubiquitination of PD-L1 at K48 which leads to the proteasomal degradation of PD-L1. Further research revealed that ES-072 activates GSK3α through the AKT pathway. GSK3α phosphorylates PD-L1 at Ser279 and Ser283, and phosphorylation of PD-L1 promotes E3 ubiquitin ligase ARIH1 to ubiquitinate PD-L1 at K48. This demonstrates that phosphorylation and ubiquitination of PD-L1 act synergistically to promote its degradation (158).

Cha et al. discovered that AMPK can directly phosphorylate PD-L1 at Ser195. This induces abnormal endoplasmic reticulum mannose trimming during PD-L1 glycosylation, preventing PD-L1 from translocating to the cell membrane. Ultimately, this may lead to a reduction in PD-L1 expression through endoplasmic-reticulum-associated degradation (159).

The post-translational regulation of PD-L1 in NSCLC primarily involves ubiquitination and phosphorylation. There is also research indicating that mTOR can regulate PD-L1 expression by inhibiting its lysosomal protein degradation (160). In other types of cancer, there are additional modes of regulation, such as glycosylation, palmitoylation, and acetylation (161–163). The multifaceted regulation of PD-L1 post-translational regulation in NSCLC requires further investigation. The PD-L1 regulation mechanisms are summarized in **Table 1**.

**Table 1:** PD-L1 regulation in lung cancer.

Regulation type		Regulators	PD-L1 level	References
Epigenetic regulation	Gene amplification	-	↑	(32–34)
	DNA methylation	TGF-β1	↑	(37)
	Histone methylation	EZH2	↓	(39)
	Histone acetylation	COP1	↓	(40)
Transcriptional regulation	Oncogenes	KRAS	↑	(43–45, 160)
		EML4-ALK	↑	(48–50)
		EGFR	↑	(52–60, 63–69)
		MYC	↑	(78–80)
		PTEN	↑	(4, 81, 84, 85)
	Signaling pathway	MAPK	↑	(86, 87, 90)
		Hippo	↑	(92–95)
		ADORA1-ATF3	↓	(96)
	Cytokines	IFN-γ	↑	(69, 99–105)
		IFN-β	↑	(106)
		HIF-1α	↑	(56, 107–109)
		TGF-β	↑	(112–114)
		IL-27	↑	(127)
	Other	MUC1	↑	(130)
Post-transcriptional regulation	microRNA	miR-34, miR-140, let-7, miR-200, and miR-200a-3p	↓	(131–137)
	Other	TTP	↑	(141)
		HuR	↓	(142)
Translational regulation		UROD	↓	(149)
		eEF2K	↑	(150).
Post-translational regulation	Ubiquitination	USP22	↑	(151) (152)
		USP8	↓	(154)
		MARCH8	↓	(155)
		CMTM6	↑	(157)
		TRAF6	↑	(154)
	Phosphorylation	GSK3α	↓	(158)
		AMPK	↓	(159)

LLC, Lewis lung carcinoma; TGF-β1, Transforming growth factor-β1; EZH2, Enhancer of Zeste homolog2; COP1, E3 ligase constitutive photomorphogenesis protein 1; KRAS, Kirsten rat sarcoma viral oncogene homologue; EML4-ALK, Echinoderm microtubule-associated protein like-4-anaplastic lymphoma kinase; EGFR, Epidermal growth factor receptor; PTEN, Phosphatase and tensin homolog; MAPK, Mitogen-activated protein kinase; IFN-γ, Interferon-γ; IFN-β, Interferon-β; HIF-1α, Hypoxia-inducible factor-1α; TGF-β, Transforming growth factor-β; IL-27, Interleukin-27; MUC1, Mucin 1; TTP, AU-rich element-binding protein tristetraprolin; HuR, Human antigen R; UROD, Uroporphyrinogen Decarboxylase; eEF2K, Eukaryotic Elongation Factor 2 Kinase; USP22, Ubiquitin-specific protease 22; USP8, Ubiquitin-specific protease 8; MARCH8, Membrane-associated RING-CH 8; CMTM6, CKLF-like MARVEL transmembrane domain containing 6; TRAF6, Tumor necrosis factor receptor-associated factor 6; GSK3α, Glycogen synthase kinase 3α; AMPK, Adenosine 5’-monophosphate-activated protein kinase. The up and down arrows, respectively, represent PD-L1 upregulation and downregulation.

### 3.2 IDO regulation

Indoleamine 2,3-dioxygenase (IDO) expression is identified as an independent negative prognostic factor in cancer (164, 165). IDO expression is associated with tumor-infiltrating forkhead box P3 positive regulatory T-cells (FoxP3+ Tregs) and is negatively associated with CD8+ cytotoxic T-cells (165). Currently, research on IDO regulation is rare. IL-27 can upregulate IDO mRNA levels and cell surface protein levels in A549 cells (127). IL-6 activates STAT3, which can bind to the IDO promoter, increasing IDO expression. The elevated IDO, in turn, promotes the transcription of IL-6 through AHR (aromatic hydrocarbon receptor), resulting in increased IL-6 secretion. This establishes an IDO–AHR–IL-6–STAT3 loop to sustain IDO expression (166). COX-2 (cyclooxygenase-2)/PGE2 (prostaglandin E2) pathway can support IDO1 constitutive expression in NSCLC; downstream β-catenin and ETV4 can bind to IDO1 promoter and promote IDO1 transcription (167).

### 3.3 GITRL regulation

Glucocorticoid-induced TNFR related protein (GITR) is expressed in various immune cells including T cells and natural killer cells. GITR can be activated by its ligand GITRL, leading to increased resistance to tumors and viral infections (168, 169). In an *in vitro* lung cancer microenvironment model, TGF-β has been shown to upregulate the expression of GITRL on DC cells (111). Further exploration is needed to command more specific details of mechanisms that can regulate GITR/GITRL in lung cancer.

## 4 Natural products targeting immune checkpoints or combined with immunotherapy

### 4.1 Natural products targeting the PD-1/PD-L1 axis

Most research focuses on natural products targeting the PD-1/PD-L1 axis in lung cancer. The strategy includes blocking the interaction between PD-1 and PD-L1 and influencing PD-1/PD-L1 expression (upregulation or downregulation).

#### 4.1.1 Natural products block PD-1/PD-L1 interaction

The interaction between PD-1 and PD-L1 can suppress the proliferation, activation, and function of CD8+ T cells (170). The combination of PD-1 and PD-L1 is reversible and the competitive binding to PD-L1 or PD-1 can inhibit the interaction between these immune checkpoints (171). The blockade of PD-1/PD-L1 interaction further restores T cells’ anti-tumor function (171).

Quercetin, a flavonoid derived from various fruits and vegetables, can inhibit progression and enhance apoptosis in various types of cancer (172, 173). Quercetin can inhibit the interaction between His-HA-PD-1 and His-HA-PD-L1 with an IC50 value of 5μM in the ELISA assay (174). Quercetin interacts with PD-L1 more strongly because the dissociation constant of quercetin to PD-L1 is smaller than that to PD-1. It can enhance PMBC cells to kill PD-L1 high-expressed NCI-H460 cells. The researcher further proved that quercetin can inhibit PD-1/PD-L1 binding in HEK293 cells and suppress MDA-MB-231 xenografted mouse tumor growth by reactivating T cells. Nevertheless, the effect of quercetin inhibiting PD-1/PD-L1 in lung cancer needs further exploration.

#### 4.1.2 Natural products downregulate tumor cell PD-L1 expression

Natural products can downregulate PD-L1 expression through different mechanisms. Most of them regulate PD-L1 at the transcriptional level, mainly through PI3K-AKT, JAK-STAT, NF-κB, and p53 signaling pathways. Some natural products can inhibit PD-L1 protein synthesis, promote PD-L1 protein degradation, and promote PD-L1 extracellular secretion to suppress PD-L1 expression (**Table 2**). All these natural products aim to inhibit tumor cells and activate immune cells.

**Table 2:** Natural products inhibit PD-L1 expression on tumor cell.

Natural product	Target/ Therapy	Cell type/ Model	Effect	Mechanism/ Pathway	Pharmacological effect	Origin	Reference
Gallic Acid	PD-L1 Combine with PD-1 mAb	H292, A549	PD-L1 mRNA and protein↓	PI3K-Akt	Tumor cell growth and survival↓ Activates the T-cell-mediated immune response Enhance PD-1 mAb	natural plants, fruits, and green tea	(175)
Silibinin	PD-L1	A549, H292, H460	PD-L1 protein↓	PI3K-Akt JAK2-STAT5	Cell Proliferation Migration and Invasion↓ Cell cycle arrest and apoptosis↑	Silybum marianum	(176)
Lycopene	PD-L1 Combine with PD-1 mAb	LLC mice	PD-L1↓	PI3K-Akt	Enhance PD-1 mAb Tumorigenesis ↓ Apoptosis ↑ CD4+/CD8+ ↑ Tumor volume ↓	Tomato	(177, 178)
Fraxinellone	PD-L1	A549, A549 mice	Cell-surface PD-L1↓	JAK1,JAK2-STAT3 mTOR MAPK	Cell proliferation/growth ↓ Tumor volume ↓	Dictamnus dasycarpus	(179)
EGCG	PD-L1	A549, H1299, Lu99 mice inject NNK	Cell-surface PD-L1 protein↓ PD-L1 mRNA and protein↓	JAK2-STAT1	Tumor growth↓ PD-L1 positive cell in mice↓ Average tumor number ↓	Green tea	(180)
Luteolin and apigenin	PD-L1 Combine with PD-1 mAb	KRAS-mutant H358, H460, H2122, A549 Nude mice- inject H358 C57bl/6J mice inject LLC	PD-L1 mRNA and protein↓	JAK-STAT3	Cell growth ↓ apoptosis↑ Enhance PD-1 mAb Tumor volume ↓ Enhance PD-1 mAb suppress tumor volume	Various fruits and vegetables	(181–183)
Myricetin	PD-L1, IDO1	A549, NCI-H1650, NCI-H460	PD-L1 mRNA and protein↓ IDO1 mRNA and protein↓	JAK-STAT	-	Ampelopsis grossedentata and Xanthoceras sorbifolia	(184)
Nobiletin	PD-L1 Combine with PD-1 mAb	A549, H292, H460	Cell-surface PD-L1 ↓ PD-L1 mRNA and protein↓	JAK2-STAT3	Tumor progression↓ Enhance PD-1 mAb	citrus peels	(185)
Oleic acid and oleoylethanolamide	PD-L1	A549	PD-L1 mRNA and protein↓	JAK-STAT1	Apoptosis↑	olive oil	(186)
Ursolic Acid	PD-L1	A549, H460	PD-L1 mRNA and protein ↓	JAK2-STAT3	Cell Proliferation↓ Cell Cycle Arrest and Apoptosis↑	fruits and medicinal herbs	(187)
Butein	PD-L1	A549, H292, H460, HCC827, H1975, H1299, PC-9, CT26	PD-L1 mRNA and protein↓ Cell-surface PD-L1 ↓	STAT3	Cell growth↓ Killing ability of T cells↑	Rhus verniciflua Stokes bark	(188)
Triptolide	PD-L1	NCI-H460, NCI-H1650	PD-L1 mRNA and protein↓ Cell-surface PD-L1 ↓	JAK1/2-STAT1/3 IRF-1	-	Tripterygium wilfordii Hook. F.	(189)
Andrographolide	PD-L1 Combine with PD-1 mAb	H1975, H1299, H1650, H460, BEAS-2B	PD-L1 mRNA and protein↓ Cell-surface PD-L1 ↓	STAT3	Cell proliferation↓ Apoptosis↑	Andrographis paniculate (Burm.f.) Nees	(190)
Ginsenoside Rg3	PD-L1	A549, A549/DDP cell (cisplatin-resistance)	PD-L1 protein↓	Akt NF-κB	Cell viability↓ Attenuated DDP resistance Apoptosis↑	Panax Ginseng	(191)
Ginsenoside Rk1	PD-L1	A549, PC9 A549 mice	PD-L1 protein↓	NF-κB	Cell growth↓ Cell apoptosis↑ Tumor volume ↓	ginseng	(192)
Arsenic sulfide	PD-L1	A549, A549/DDP, A549/DDP mice	PD-L1↓	p53/miR-34a-5p axis	Sensitivity of to DDP↑ Apoptosis↑ Tumor volume ↓	Realgar	(193)
Evodiamine	PD-L1 Combine with PD-1 mAb	H1975, H1650 H1975 or LLC mice	PD-L1 mRNA↓ Cell-surface PD-L1 ↓	through MUC1-C/PD-L1 axis	Cell growth↓ Apoptosis↑ *in vitro* tumor volume ↓ Enhance PD-1 mAb suppress tumor volume Improving survival	Tetradium	(194)
Nemania sp. EL006872 extract (mainly radianspenes C and D, and dahliane D)	PD-L1, ICOSL, GITRL	H1975	PD-L1 mRNA↓ Cell-surface PD-L1 ↓ GITRL, and ICOSL↓	Inhibit PD-L1 expression through AHR	Suppress cancer cell proliferation	endolichenic fungus	(195)
Licochalcone A	PD-L1, IDO1	A549, H1299, H1650	Cell-surface PD-L1 ↓ IDO1 protein↓	Inhibition PD-L1 protein synthesis	-	Glycyrrhiza inflata Batalin	(196)
Berberine	PD-L1	A549, H157, H358, H460, H1299, H1975, LLC mice	Cell-surface PD-L1 ↓ PD-L1 protein↓	Ubiquitinates PD-L1 to induce protein degradation	Sensitivity of cancer cells to T-cell↑ Tumor growth↓ Tumor volume ↓	Coptis chinensis	(197)
Berberine	PD-1/PD-L1	Urethane-Induced Lung Model	Serum PD-L1 level↓ Spleen T lymphocyte surface PD-1 expression↓	-	Attenuate immunosuppressive NLR level↓	Coptis chinensis	(198)
SA-49	PD-L1	A549, NCI-H157, NCI-H1975, NCI-H1299, NCI-H460, Lewis cells	PD-L1 protein↓	Induce PD-L1 lysosomal degradation	Enhances the cytotoxicity of T and NK cells Tumor volume ↓	Sophora alopecuroides L.	(199)
Platycodin D	PD-L1	NCI–H1975, NCI–H358	Cell-surface PD-L1 ↓ PD-L1 protein↓	Triggers the extracellular release of PD-L1	Restore the Jurkat T cells activation	Platycodon grandiflorus (Jacq.) A. DC.	(200)
Usnic acid	PD-L1	A549	PD-L1 protein↓	-	-	Usnea diffracta Vain	(201)
Lac water extract (active components laccaic acid A, B, C, and E)	PD-L1	PC9, A549	PD-L1 protein↓	-	-	Kerria lacca	(202)
Acorus calamus L. Polysaccharide	PD-L1	LLC mice	PD-L1↓	-	Inhibit tumor growth Tumor volume ↓	Acorus calamus L.	(203)

LLC, Lewis lung carcinoma; NNK, 4-(methylnitrosamino)-1-(3-pyridyl)-1-butanone; AHR, aromatic hydrocarbon receptor. The up and down arrows, respectively, represent upregulation and downregulation.

Gallic acid, silibinin, and lycopene can inhibit PD-L1 expression through PI3K-AKT signaling. Gallic acid is derived from fruits, plants, and green tea (204, 205). It can prevent carcinogenesis, inhibit cancer cell proliferation, and induce cancer cell apoptosis (206, 207). Gallic acid can inhibit PD-L1 protein and mRNA levels in A549 and H292 cells. Further study proves that gallic acid binds to and therefore inhibits EGFR phosphorylation, binging about PI3K/AKT phosphorylation reduction and its downstream p53 protein and mRNA expression increase, and finally, through miR-34a to downregulate PD-L1 expression (175). Silibinin is extracted from Silybum marianum, a plant traditionally used to treat liver diseases; it can suppress tumorigenesis in various cancer types and inhibit lung cancer proliferation and angiogenesis (208, 209). Alexis et al. proved that silibinin inhibits NSCLC cell PD-L1 expression by suppressing PI3K/AKT molecular phosphorylation, and silibinin also influences PD-L1 expression through JAK2/STAT5 signaling (176). Lycopene, mainly present in tomatoes, can block AKT signaling by inhibiting AKT phosphorylation from downregulating IFN-γ induced PD-L1 protein and mRNA expression in LLC cells (177, 178). Epidermal growth factor receptor (EGFR) is the main receptor influencing PI3K-AKT signaling; gallic acid and silibinin can bind to EGFR and inhibit its phosphorylation, influencing the downstream signaling pathway (175, 176).

Fraxinellone, EGCG, luteolin, myricetin, oleic acid, silibinin, ursolic acid, butein, triptolide, and andrographolide can downregulate the PD-L1 expression through the JAK-STAT signaling pathway. D. dasycarpus is a traditional herb with various medical functions (210–212). Fraxinellone, a limonoid extracted from D. dasycarpus, can suppress the phosphorylation of JAK1 and JAK2 and then inhibit STAT3 transcription and phosphorylation, and it also inhibits HIF-1α protein synthesis; both pathways can reduce PD-L1 expression (179). (–)-epigallocatechin gallate (EGCG) is the main polyphenol in green tea whose cancer prevention effect has been widely reported (213). EGCG can reduce the p-STAT1 level, and further analysis showed that the reduction of p-STAT1 is highly correlated to the down-regulation of cell-surface PD-L1. EGCG can also influence the PI3K-AKT pathway, but this pathway failed to reduce surface PD-L1 (180). Luteolin can be discovered in various fruits and vegetables (181, 182). Luteolin and its derivative, apigenin, can potentially enhance chemotherapy (214, 215). Jiang et al. discovered that apigenin and luteolin inhibit cell surface PD-L1 and PD-L1 mRNA expression through downregulating STAT3 phosphorylation (183). Myricetin, also derived from various fruits and vegetables, possesses immune regulation and anti-tumor effects (216–221). Chen et al. discovered that myricetin inhibits PD-L1 protein and mRNA expression by inhibiting IFN-γ induced STAT1 and STAT3 phosphorylation (184). Nobiletin is from citrus peels that induce apoptosis function in lung cancer (222). Nobiletin downregulated the phosphorylated EGFR, JAK2, and STAT3 to suppress cell-surface protein and mRNA levels of PD-L1. Nobiletin can also promote miR-197 expression, inhibiting the STAT3 pathway (185). Oleic acid is an extract of olive oil and can be transformed in the gut and become oleoylethanolamide (OEA) (223, 224). Oleic acid has an anti-tumor effort, and oleic acid and OEA can block IFN-γ induced STAT1 phosphorylation to suppress PD-L1 protein and mRNA expression (186). Ursolic acid, derived from many fruits and herbs, inhibits cancer cell proliferation and autophagy (225–227). Ursolic acid can bind to EGFR and block its activation, suppressing downstream JAK2 and STAT3 phosphorylation. STAT3 can bind to the CD274 promoter and promote PD-L1 mRNA synthesis, thus upregulating PD-L1 protein expression (187). Butein is derived from Rhus verniciflua Stokes bark, and it can inhibit tumor angiogenesis, invasion, and metastasis (228, 229). Butein can inhibit STAT1 transcription and downregulate IFN-γ induced PD-L1 expression (188). Triptolide is extracted from Tripterygium wilfordii Hook. F., and Triptolide has been proven to regulate PD-L1 in other tumor cell lines, such as breast cancer and glioma (230–232). Triptolide can inhibit STAT3 to inhibit IFN-γ induced PD-L1 expression; it also inhibits JAK1 and JAK2 expression, STAT1 phosphorylation, and IRF1 protein level, resulting in PD-L1 transcriptional inhibition (189). Andrographolide (AD), extracted from Andrographis paniculate (Burm.f.) Nees (A. paniculate), was reported to possess anti-inflammatory and anti-cancer characteristics (233–235). AD can downregulate IFN-γ induced PD-L1 expression through downregulating the phosphorylation of STAT3 (190).

Ginsenoside Rg3 and Rk1 can downregulate PD-L1 expression through the NF-κB signaling pathway and Arsenic sulfide through the p53 signaling pathway. Panax Ginseng is widely used in traditional Chinese medicine to enhance immunity (236, 237). Ginsenoside Rg3 and Rk1 derived from Panax Ginseng can attenuate PD-L1 expression. Rg3 and Rk1 inhibit the phosphorylation of p65, while Rk1 also inhibits p65 expression and IKKα and IκBα phosphorylation, and Rg3 can also affect PI3K-Akt signaling (191, 192). Realgar derivative Arsenic sulfide (As_4_S_4_) has anti-tumor activities in several cancers (238, 239). It suppresses PD-L1 protein expression by upregulating p53 and miR-34a-5p levels (193).

Evodiamine and Nemania sp. EL006872 can downregulate PD-L1 expression through other mechanisms. Evodiamine is derived from Tetradium with anti-tumor activity (240, 241). MUC1-C can upregulate PD-L1 expression, and Jiang et al. discovered that evodiamine downregulates MUC1-C mRNA and protein expression and correlates with PD-L1 suppression (194). Nemania sp. EL006872 is an endolichenic fungi isolated from lichen Bryoria fuscescens (Gyelnik) Brodo and D. Hawksw; its secondary metabolites (mainly radianspenes C and D, and dahliane D) downregulate the aromatic hydrocarbon receptor (AHR) therefore decreasing surface PD-L1 expression in benzo[a]pyrene treated H1975 cells (195).

Licochalcone A, berberine, and SA-49 can influence protein synthesis or degradation of PD-L1. Licochalcone A (LCA) is derived from the root of Glycyrrhiza inflata Batalin. LCA induces apoptosis and decreases viability in lung cancer cells. That LCA abolishes PD-L1 protein synthesis induced by IFN-γ was proved by Yuan et al. using the Click-iT protein synthesis assay (196). Berberine is derived from the traditional Chinese medicine Coptis chinensis and has an anti-tumor effect with less cytotoxicity to normal cells (242, 243). Berberine can ubiquitinate PD-L1 and induce PD-L1 protein degradation without influencing PD-L1 transcription (197). Platycodin D (PD), a natural product isolated from Platycodon grandiflorum (244), decreases lung cancer cell surface PD-L1 levels by triggering PD-L1 extracellular release (200). Aloperine, derived from Sophora alopecuroides L., possesses an anti-tumor effect (245, 246). And its derivate, SA-49, was discovered can trigger PD-L1 degradation (199). Further exploration showed SA-49 inhibits GSK3β activation, inactivated GSK3β enhanced MITF transcription activity, and MITF finally promoted lysosome biogenesis, triggering PD-L1 lysosomal degradation (199).

Usnic acid derived from Usnea diffracta Vain and Lac water extract (active components laccaic acid A, B, C, and E) derived from lac insects can downregulate PD-L1 protein expression in lung cancer cells (201, 202, 247). While the researchers further explore related pathways in other tumor cells, such as Hela cells and melanoma cells, the mechanism of these natural products regulating PD-L1 expression in lung cancer needs further exploration. Acorus calamus L. Polysaccharide can suppress PD-L1 levels in LLC mice but also needs further exploration of the mechanisms (203).

#### 4.1.3 Natural products upregulate tumor cell PD-L1 expression

PD-L1 expression in tumor cells is closely related to the effectiveness of anti-PD-L1 therapy; thus, some researchers sought to find natural products that increase PD-L1 expression (**Table 3**) (254).

**Table 3:** Natural products promote PD-L1 expression on tumor cell.

Natural product	Target/ Therapy	Cell type/ Model	Effect	Mechanism/ Pathway	Pharmacological effect	Origin	Reference
Sophocarpine	PD-L1 Combine with ICI	H1975, A549, LLC LLC mice	PD-L1 protein ↑	ADORA1-ATF3	Tumor volume ↓ Enhance anti-PD-L1 immunotherapy suppress tumor volume	Sophora alopecuroides Linn	(248)
Nagilactone E	PD-L1	A549, NCI-H460, NCI-H1975	PD-L1 mRNA and protein ↑ Cell-surface PD-L1↑	JNK-c-Jun	May improve the PD-1/PD-L1 antibody therapies	Podocarpus nagi (Thunb.) Pilg	(249)
Resveratrol	PD-L1	A549, H1299	PD-L1 mRNA and protein ↑	Wnt pathway	Suppressed T cell function	in fruits and vegetables.	(250)
Resveratrol	PD-L1	A-427, A549, HCC827, NCI-H1299, NCI-H1581, NCI-H1703, NCI-H358	PD-L1 mRNA and protein ↑	-	Antiproliferative activity against all the lung cancer cell	in fruits and vegetables.	(251)
Z-guggulsterone	PD-L1	A549, H1975, LLC LLC mice	PD-L1 mRNA and protein ↑ Cell-surface PD-L1↑	Partly through FXR inhibition Partly by the activation of the Akt and Erk1/2 signaling pathways	Inhibits cell viability Tumor volume ↓	Commiphora mukul tree	(252)
Fascaplysin	PD-L1 Combine with PD-1 mAb	A549 and LLC cell	PD-L1 protein↑	-	Promote CD4+ and CD8+ T cell infiltration when combined with PD-1 mAb tumor volume ↓ Enhance anti-PD-L1 immunotherapy suppress tumor volume	Sponges	(253)

LLC, Lewis lung carcinoma; FXR, farnesoid X receptor. The up and down arrows, respectively, represent upregulation and downregulation.

Sophora alopecuroides Linn (SAL) is a Chinese herb with immunomodulating and anti-tumor functions (255). Sophocarpine (Sop) is one of the Alkaloids from SAL, and it can upregulate ADORA1 and ATF3 expression to increase the mRNA and protein level of PD-L1 (248).

Nagilactone E (NLE) is a diterpenoid derived from Podocarpus nagi seed oil—which can inhibit NSCLC cell migration and invasion (256, 257). NLE can promote the expression and phosphorylation of JNK to phosphorylate c-Jun and then upregulate mRNA, protein, and membrane PD-L1 expression (249).

Resveratrol is a polyphenol from fruits and vegetables. Studies have found that resveratrol possesses anti-proliferation, anti-migration, and enhanced chemotherapy properties. Resveratrol can inhibit Axin2 transcription, destabilize β-catenin, and promote β-catenin/TCF binding to CD274 promoter, thus upregulating PD-L1 expression (250).

Z-guggulsterone (Z-GS), extracted from the gumresin of the Commiphora mukul tree, can reduce growth and induce apoptosis in cancer cells (258, 259). Z-GS can inhibit the farnesoid X receptor, a transcription factor that inhibits PD-L1 expression, which is the partial reason Z-GS promotes PD-L1 expression (260). Z-GS can also activate PI3K/AKT (promote AKT phosphorylation) and MEK/Erk1/2 (promote ERK phosphorylation) pathways to induce PD-L1 expression (252).

Sponges extractive fascaplysin can upregulate PD-L1 expression and enhance anti-PD-1 immunotherapy in NSCLC cells and LLC tumor-bearing mice (253, 261). Fascaplysin combined with anti-PD-1 immunotherapy promoted CD4+ and CD8+ T cell infiltration. However, the mechanism fascaplysin influences PD-L1 remains unclear (253).

#### 4.1.4 Natural products influence PD-1 or PD-L1 on immune cells

Some natural products can influence PD-1 or PD-L1 expression on immune cells, providing another anti-tumor method (**Table 4**).

**Table 4:** Natural products influence immune cells PD-1 or PD-L1 expression.

Natural product	Target/ Therapy	Cell type/ Model	Effect	Mechanism/ Pathway	Pharmacological effect	Origin	Reference
β-glucan	PD-L1	LLC mice	Tumor-educated dendritic cells surface PD-L1 ↓	-	Tumor volume ↓ Abrogated immune suppression CD4+PD-1+ T cells both in the tumor and the draining lymph nodes↓	from the cell walls of Saccharomyces cerevisiae.	(262)
Cordyceps militaris polysaccharide	PD-L1	LLC mice	M2 macrophages PD-L1↓	-	Anti-tumor effect Tumor volume ↓	Cordyceps militaris	(263)
Tussilago farfara L. Polysaccharides	PD-L1	LLC mice	Decrease peripheral CD274+ lymphocytes	-	-	Tussilago farfara L.	(264)
Platycodon grandiflorum	PD-1 Combine with ICI	LLC, H1975, A549 LLC mice	PD-1 on CD8+ T cells ↓	Indirectly reduces PD-1 on CD8+ T cells through the VEGF-A–VEGFR-2 axis in tumor	Tumor growth ↓ Tumor volume ↓ CD8+ T cell infiltration↑ Improving survival	Platycodon grandiflorum	(265)
Artesunate	PD-1	Patient	CD8+、CD4+ T cell PD-1↑	-	-	Artemisia carvifolia	(266)

LLC, Lewis lung carcinoma. The up and down arrows respectively represent upregulation and downregulation.

Tumor-educated DCs can inhibit CD4(+) T cell proliferation and suppress T cell response, β-glucan can downregulate tumor-educated dendritic cells surface PD-L1 expression in LLC mice, which enhances priming of Th1 cells and CD8+ T cells while decreasing Treg cell differentiation (262, 267). Cordyceps militaris polysaccharide can inhibit M2 macrophage PD-L1 expression, induce polarization of M2 macrophages to M1, and reverse the immunosuppression effect of M2 macrophages on T lymphocytes (263). Tussilago farfara L. Polysaccharides can decrease peripheral CD274+ lymphocytes in LLC mice (264).

Platycodon grandiflorum (PG) is used as medicine for pulmonary and respiratory diseases in traditional Chinese medicine, and its immunomodulatory and anti-cancer effects were widely reported (244, 268–272). Unlike other natural products targeting PD-L1 expression on tumor cells, PG can reduce PD-1 expression on the CD8+ T cell surface. Yang et al. discovered that PG could reduce STAT3 phosphorylation and downregulate VEGF-A secretion in tumor cells. VEGF-A-VEGFR axis can upregulate PD-1 expression on CD8+ T cells; thus, PG indirectly inhibits CD8+ T cells PD-1 by inhibiting tumor VEGF-A secretion (265).

Artesunate is extracted from Artemisia carvifolia. Xing et al. discovered that Artesunate can upregulate PD-1 expression on peripheral CD8+ or CD4+ T cells. Moreover, they discovered that age over 60, lymphocytes>1.26×10^9^/L, neutrophil to lymphocyte ratio (NLR)<5, chemotherapy, and monocytes between 0.29-0.95×10^9^/L are positive factors that promote Artesunate induce CD8+ cell PD-1 expression. Lymphocytes ≤ 1.26×10^9^/L, NLR<5, chemotherapy, monocytes between 0.29-0.95×10^9^/L, and TNM stage≤III are positive factors that promote Artesunate inducing CD4+ cell PD-1 expression. Artesunate-induced PD-1 expression in CD8+ or CD4+ T cells may improve the prognosis of patients undergoing immune checkpoint inhibitor therapy (266). As mentioned, berberine also downregulates spleen T lymphocyte surface PD-1 expression and reverses the doxorubicin-induced immunosuppressive microenvironment (197).

### 4.2 Natural products targeting other immune checkpoints

Natural products that target other immune checkpoints are listed in **Table 5**. Astragaloside IV, erianin, dihydroartemisinin, calotropin, and Berbamine Hydrochloride can target other immune checkpoints besides PD-1 and PD-L1. Tumor-expressed IDO can limit the anti-tumor function of cytotoxic T cells (278). Astragalus membranaceus is a widely used herb in traditional Chinese medicine with strong immune modulatory properties (279, 280). Astragaloside IV extracted from Astragalus membranaceus can inhibit IDO expression in IDO-overexpressed lung cancer cells, upregulate co-cultured CD8+CD28+ cells, and downregulate Treg cells (273). Erianin extracted from the Dendrobium Chrysostom also can downregulate IDO mRNA and protein levels (274, 281). Dihydroartemisinin (DHA), derived from the antimalarial drug Artemisia annua, can downregulate B7-H3 expression (275, 282). Calotropin isolated from Asclepias curasavica L can repress CTLA-4 expression (276, 283). Berbamine Hydrochloride is derived from Coptis Chinensis and has an anti-tumor function (284–286). Berbamine Hydrochloride can activate Nox2 to resist lysosomal acidification and, therefore, inhibit lung cancer cell autophagy, which may potentiate the effect of chemotherapy (277).

**Table 5:** Natural products influence other immune checkpoints.

Natural product	Target/ Therapy	Cell type/ Model	Effect	Mechanism/ Pathway	Pharmacological effect	Origin	Reference
Astragaloside IV	IDO	IDO-overexpressed LLC mice	IDO mRNA and protein↓	-	Percentage of Tregs↓ Percentage of CTLs in spleen↑ Tumor volume ↓ Improving survival	Astragalus membranaceus	(273)
Erianin	IDO	2LL	IDO mRNA and protein ↓	-	Cell proliferation↓ Cell invasion, metastasis, and angiogenesis↓	Dendrobium Chrysostom	(274)
Dihydroartemisinin	B7-H3	A549, HCC827 A549 transplanted nude athymic mice	B7-H3 protein ↓	-	Cell growth and migration↓ apoptosis↑ Tumor growth↓ Tumor volume ↓	Artemisia annua	(275)
Calotropin	CTLA-4	H358 H358 transplanted nude mice	CTLA-4 protein ↓	-	Cell growth, migration and invasion↓ Apoptosis↑ Tumor volume ↓ Improving survival	Asclepias curasavica L	(276)
Berbamine Hydrochloride	NOX2	nude mice xenograft H1299, H1975	Nox2 activation	Resists lysosomal acidification by activating Nox2	Enhance killing effect of chemotherapy Tumor volume ↓	Coptis Chinensis (Ranunculaceae)	(277)

LLC, Lewis lung carcinoma. The up and down arrows, respectively, represent upregulation and downregulation.

Some other natural products that were described before targeting the PD-1/PD-L1 axis also affect other immune checkpoints: Nemania sp. EL006872 extract can downregulate GITRL and ICOSL protein expression (195); Licochalcone A can downregulate IDO1 protein expression (196).

### 4.3 Natural products combine with other therapies targeting immune checkpoints or combine with immunotherapy

Natural products can combine with other therapies like radiotherapy and chemotherapy and modulate immune checkpoint expression. Natural products can combine with immune checkpoint inhibitors to enhance the immunotherapy effect (**Table 6**).

**Table 6:** Natural products combined with other therapy.

Natural product	Target/ Therapy	Cell type/ Model	Effect	Mechanism/ Pathway	Pharmacological effect	Origin	Reference
Fuzi	PD-L1 Combine with radiation	LLC mice	Reduce radiation induced PD-L1 mRNA and protein expression	-	Enhance efficacy of irradiation Tumor volume ↓ Improving survival	Aconitum carmichaelii Debx. (Ranunculaceae)	(287)
Astragaloside IV	B7-H3 Combine with Cisplatin	A549, HCC827, H1299	B7-H3 mRNA and protein ↓	-	-	Astragalus membranaceus	(288)
Ginsenoside Rh2	PD-L1 Combine with Cisplatin	A549, H1299	Reduce cisplatin induced PD-L1 mRNA and protein expression	EGFR-PI3K-AKT-mTOR	Enhanced Cisplatin-Induced A549 Cell Death, Apoptosis	Panax ginseng C.A. Meyer (Araliaceae)	(289)
Silibinin	PD-L1 Combine with crizotinib	NCI-H3122 crizotinib resistance cell	PD-L1 mRNA↓	JAK-STAT3	Reverses acquired resistance to crizotinib	Silybum marianum	(290)
Astragalus Polysaccharide Injection	Combine with ICI	Patient	-	-	Decrease NLR	Not mentioned	(291)
Cryptotanshinone	Combine with ICI	A549, LLC LLC mice	-	-	Can eliminate tumor tumor volume ↓ CD4+ and CD8+ T cell↑, DCs↓	Danshen/Salvia miltiorrhiza Bunge	(292)

LLC, Lewis lung carcinoma. The up and down arrows, respectively, represent upregulation and downregulation.

Aconitum carmichaelii Debx, also called Fuzi in traditional Chinese medicine; previous studies proved that Fuzi inhibits tumor growth by improving immune responses (293, 294). Radiotherapy increases lung cancer tissue PD-L1 mRNA and protein levels, while Fuzi decreases it. Furthermore, when Fuzi combines with radiotherapy, it can increase serum immune-promoting cytokine (IL-2, IL-5, IL-6, IL-12) levels but decrease immune-suppressing cytokine (IL-10, TGF-β) levels (287).

B7-H3 is an immune checkpoint overexpressed on tumor cells and is positively related to poor prognosis and tumor progression (295, 296). Astragaloside IV sensitizes lung cancer cells to cisplatin by decreasing B7-H3 mRNA and protein levels (288).

Ginsenoside Rh2 and Rg3, both extracted from ginseng, can downregulate lung cancer cells PD-L1 expression induced by cisplatin: Ginsenoside Rh2 through PI3K/Akt pathway while Rg3 through PI3K/Akt and NF-κB pathway (191, 289). Moreover, Rh2 and Rg3 can attenuate cisplatin resistance and promote apoptosis in lung cancer cells.

Astragalus Polysaccharide Injection (PG2) is a prescription drug that alleviates cancer-related fatigue (297). Shih et al. treated lung cancer patients with immune checkpoint inhibitors (ICIs) and PG2 or ICIs alone and found out that PG2 can decrease the patient neutrophil-to-lymphocyte ratio (NLR). A low NLR is correlated with a better prognosis (291). Salvia miltiorrhiza Bunge (Danshen) extract cryptotanshinone, combined with anti-PD-L1 antibody, can make LLC-bearing mice tumor-free and increase CD45+ leukocytes, CD4+, and CD8+ T cells infiltration in tumors (292, 298). Some natural products, such as Platycodon grandiflorum and Sophocarpine, have been reported to enhance the efficacy of anti-PD-L1 antibodies, suppressing tumor growth (248, 265). Evodiamine has shown the ability to enhance the effectiveness of anti-PD-1 monoclonal antibodies (mAb), suppressing tumor growth and improving survival in LLC mice (194). Luteolin and apigenin have been observed to augment the impact of monoclonal antibodies, leading to tumor growth suppression, enhanced survival in LLC mice, and a notable increase in CD8+ T cells within the blood, spleen, and tumor (183). Nobiletin has demonstrated its ability to enhance PD-1 mAb treatment, promoting cytokine secretion in peripheral blood mononuclear cells (PBMC) and inducing greater tumor cell death (185). Lycopene has been shown to enhance the impact of PD-1 mAb therapy, resulting in increased tumor cell death, elevated production of anti-tumor cytokines (IL-2, IFNγ), and a higher CD4+/CD8+ ratio in the spleen (178). Gallic acid can potentially enhance the impact of PD-1 mAb treatment, leading to increased tumor cell death in a PBMC co-cultured system and elevated PBMC IFN-γ secretion (175). Andrographolide has been found to potentiate the anti-tumor effect of PD-1 mAb (190).

## 5 Traditional medicine targeting immune checkpoints or combined with immunotherapy

Traditional medicine can also influence immune checkpoints or combine with immunotherapy (**Table 7**, **Supplementary Table 1**). Bu Fei decoction is widely used in improving lung function, especially in the Qi deficient condition. Bu Fei decoction can reduce mRNA and protein levels of PD-L1 in tumor cells and reduce PD-L1 protein expression in xenograft mouse lung cancer cells (299). HYR-2 (Huoxue Yiqi recipe-2) is formed from the traditional Chinese medicine Ze Qi decoction. HYR-2 downregulates PD-L1 mRNA and protein expression in H1975 cells and LLC-xenograft mice, and further exploration shows HYR-2 inhibits PD-L1 through PI3K/Akt signaling (300). Qingfei Jiedu decoction is used as a decoction treating lung cancer (310). Pan et al. used Qingfei Jiedu decoction-containing serum to treat A549 cells and observed that it could reduce cell PD-L1 expression by modulating EGFR, HIF-1, JUN, and NF-κB expression. Moreover, Qingfei Jiedu decoction increases CD8+PD-1+ T cells, which are regarded as activated T cells, in LLC-bearing mice spleen (301). Qiyusanlong decoction can inhibit PD-L1 mRNA and protein expression in tumor tissue and PD-1 mRNA and protein expression in the spleen in LLC-bearing mice (302). The Feiji Recipe can reduce spleen Treg cells in mice-bearing LLC transfected with IDO and prolong mouse life span, and further research found that Feiji Recipe can downregulate IDO in LLC mice (303, 304). Yu-Ping-Feng (YPF), an ancient Chinese herbal formula, can inhibit IDO expression, increase NK cell tumor infiltration, and enhance NK cell cytotoxicity (305).

**Table 7:** Traditional medicines influence immune checkpoints or combine with immunotherapy.

Studied Materials	Target/ Therapy	Cell type/ Model	Effect	Mechanism/ Pathway	Pharmacological effect	Reference
Bu Fei Decoction	PD-L1	M2-polarized TAM A549 or NCI-H1975 mice	PD-L1 mRNA and protein ↓	-	Suppress NSCLC cell proliferation Tumor volume ↓	(299)
HYR-2	PD-L1	A549, H1975, H1299, 95-D LLC mice	PD-L1 mRNA and protein↓	PI3K-Akt	Inhibits the proliferation of lung cancer cells *in vitro* Tumor volume ↓	(300)
Qingfei Jiedu decoction	PD-1/PD-L1	A549, LLC LLC mice	PD-L1 mRNA ↓ PD-L1 in tumor tissue↓ CD8+PD-1+T% in spleen↑	EGFR, HIF-1, JUN, and NFκB signaling	-	(301)
Qiyusanlong decoction	PD-1/PD-L1	C57BL/6 mice + LLC cell	PD-L1 mRNA and protein in tumor tissue ↓ PD-1 mRNA and protein in spleen tissue ↓	-	Tumor volume ↓	(302)
Feiji Recipe	IDO	LL/2-EGFP-IDO mice	IDO protein↓ Treg cell ratio↓	-	Tumor volume ↓ Improving survival Downregulate Treg cell Inhibit T-cell apoptosis	(303, 304)
Yu-Ping-Feng	IDO	LLC mice	IDO mRNA ↓	-	Tumor volume ↓ Improving survival Increase NK cell tumor infiltration Enhanced NK cell cytotoxicity	(305)
Lung Cancer Fang No. 1	Combine with ICIs (nivolumab, pembrolizumab, sintilimab, atezolizumab)	Patient	-	-	CYFRA21-1, CA125, and VGEF ↓ CD4+, CD3+, and CD4+/CD8+ ↑	(306)
Jianpichuji Fang	Combine with sintilimab and chemotherapy (carboplatin + pemetrexed or paclitaxel-albumin)	Patient	-	-	KPS score ↑ Incidence of digestive system nausea symptoms and leukopenia↓	(307)
Yanghe Decoction	Combine with ICI (pembrolizumab, camrelizumab, tislelizumab, and sintilimab)	Patient	-	-	CEA and CA19-9↓, stable KPS scores, relieve clinical symptoms (cough, sputum, hemoptysis, fatigue)	(308)
Warming Spleen and Kidney Fang	Combine with ICI (tislelizumab, and sintilimab)	Patient	-	-	KPS score ↑ Relieve ICIs-associated diarrhea symptoms	(309)

LLC, Lewis lung carcinoma. The up and down arrows respectively represent upregulation and downregulation.

Lung Cancer Fang No. 1 was reported to improve immune function in SCLC patients. Lung Cancer Fang No. 1 combined with PD-1/PD-L1 inhibitor chemotherapy compared with using PD-1/PD-L1 inhibitor chemotherapy can decrease CYFRA21-1, CA125, and VGEF levels, increase CD4+, CD3+, and CD4+/CD8+ levels, and improve clinical symptoms (including cough, expectoration of sputum, hemoptysis, chest tightness, fatigue, chest pain, and fever) in SCLC patients (306). A clinical study on Jianpichuji Fang in combination with ICIs for the treatment of advanced NSCLC was conducted (307). The control group received sintilimab injection in combination with platinum-based combination chemotherapy, while the experimental group received Jianpichuji Fang in addition to the treatment used in the control group. There was no significant difference in disease control rates between the two groups after two treatment cycles. However, patients in the experimental group showed a greater improvement in KPS scores compared to the control group. Moreover, the incidence of digestive system nausea symptoms and leukopenia was lower in the experimental group than in the control group. In another advanced NSCLC clinical study, Yanghe Decoction was used in combination with ICIs (308). The control group received chemotherapy combined with immune checkpoint inhibitors (including pembrolizumab, camrelizumab, tislelizumab, and sintilimab), and the treatment group received Yanghe Decoction in addition to the treatment used in the control group for a total of four treatment cycles. The treatment group demonstrated better improvement in clinical symptoms (cough, sputum, hemoptysis, fatigue), stable KPS scores, and reduced levels of tumor markers CEA and CA19-9. In a study focused on NSCLC patients experiencing diarrhea after ICI treatment, the control group received oral prednisolone (1 mg/kg), while the treatment group received Warming Spleen and Kidney Fang for a total of 4 weeks (309). The results showed no significant difference in efficacy between the control and treatment groups. Nevertheless, the KPS level increased more in the treatment group, and there were fewer occurrences of common side effects associated with hormone treatment, such as nausea, vomiting, dizziness, headache, dry mouth, throat dryness, and infection.

## 6 Conclusion and outlook

**Figure 2:**
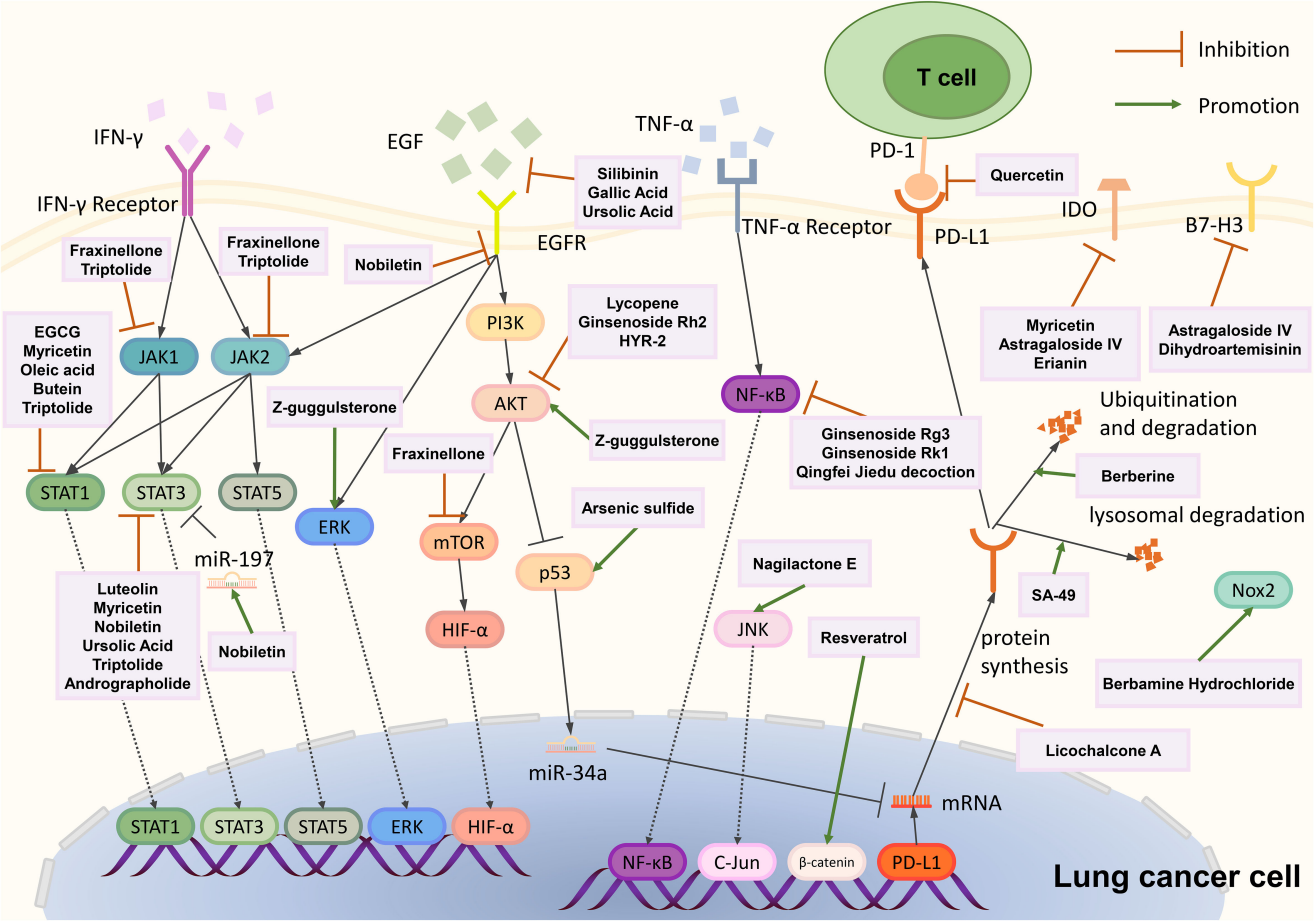
Schematic represents natural products that influence immune checkpoints in the lung cancer cell. Most natural products influence PD-L1 expression (upregulate or downregulate) through different pathways, and some natural products influence other immune checkpoints like IDO or B7-H3. Arrows indicate activations; blunt-ended lines indicate inhibitory effects; dotted arrows indicate translocations.

This study reviewed immune checkpoints regulation mechanisms and summarized natural products and traditional Chinese medicine that influence immune checkpoints or immunotherapy in lung cancer. Immune checkpoint regulation in lung cancer includes epigenetic, transcriptional, post-transcriptional, translational, and post-translational regulation. Most research focuses on PD-L1 regulation. Furthermore, transcriptional regulation was mainly studied; oncogenes or cytokines through multiple signal pathways finally influence CD274 promoter or enhancer. Exploring the PD-L1 regulator provides a basis for investigating how natural products influence PD-L1. Therefore, most current research focuses on natural products that influence the PD-1/PD-L1 axis (**Figure 2**). Some natural products influence other immune checkpoints like IDO, B7-H3, CTLA-4, GITRL, ICOSL, and Nox2, but the underlying mechanisms need further elucidation. Natural products’ immune checkpoint regulation properties provide the potential to become new clinical immune checkpoint inhibitors or as a prodrug in research (199). Natural products and Traditional medicine can influence the expression of immune checkpoints through different signaling pathways, some of which can influence multiple pathways at the same time, such as silibinin, ginsenoside Rg3, fraxinellone, Z-guggulsterone, and Qingfei Jiedu decoction (176, 179, 191, 252, 301). Moreover, myricetin, Nemania sp. EL006872, and Licochalcone A can influence multiple immune checkpoints (184, 195, 196). When combined with immunotherapy, natural products and Traditional medicine can enhance the growth inhibition ability of immunotherapy and promote anti-tumor lymphocyte infiltration (178, 194, 253, 292, 306). Traditional medicine can also alleviate immune checkpoint-related adverse reaction and enhance patients’ physical well-being (306–309). Furthermore, some natural products can inhibit immunosuppressive factor secretion: IL-10, TGF-β; promote anti-tumor factor secretion: IL-2, IL-5, IL-6, IL-12; reduce immunosuppressive immune cells: Treg, M2 microphage; increase anti-tumor CD8+ T-cells (253, 262, 263, 265, 273, 287, 292, 303, 304). As a result, the combination of immune checkpoint inhibitors with low-toxicity natural products is a good solution to treat lung cancer.

In the future, further exploring PD-L1 and other immune checkpoint regulation mechanisms and explaining the mechanisms of natural products influencing signaling pathways are needed: Silibinin alone can inhibit PD-L1 expression through both PI3K/AKT and JAK2/STAT5 signaling, while when it combines with crizotinib treating crizotinib-resistant cells, it inhibits PD-L1 expression through JAK-STAT3 signaling; Platycodon grandiflorum can reduce tumor cells downregulate VEGF-A secretion by inhibiting STAT3 phosphorylation, although it can inhibit STAT3 phosphorylation, the researcher did not report whether Platycodon grandiflorum can reduce tumor cell PD-L1 expression through this pathway (176, 265). Determining the synergistic effect among natural products influences different pathways is helpful for drug combinations.

Moreover, the low water solubility of some natural products will hinder their absorption and reduce their bioavailability in the body (311). Tumor heterogeneity is ubiquitous among the different pathological types of lung cancer, and the same natural products may have different effects on different types of lung cancer. Because of the above questions, no natural products are now used as single immune checkpoint inhibitors, and few clinical trials combine natural products with immune checkpoint inhibitors. Based on these unanswered questions, there is still a gap in the clinical use of natural products. The good news is that chemical or enzymatic modification of natural products may improve solubility and combine with a delivery system such as nanoparticles to improve drug bioavailability (312). Currently, there are numerous ongoing trials investigating natural products as lead compounds in anticancer formulations (313). Moreover, since gut microbiota can regulate the metabolism of oral natural products, understanding how gut microbiota affect each natural product also remains a critical question for the clinical use of natural products (314, 315). The extraction of natural products must adhere to principles of sustainability and cost-effectiveness. Some natural products, like ginsenoside Rk1, Rh2, and Rg3 derived from ginseng, have relatively high raw material costs and low extraction yields, limiting their widespread application. Therefore, improving the extraction yield of natural products or exploring chemical synthesis and semi-synthesis will be crucial directions for future research in this field (316).

Meanwhile, traditional medicine, a promising way for the clinical application of natural products, always combines many herbs to treat lung cancer based on clinical experience. Traditional medicine may have synergistic effects that can improve therapeutic efficiency, but the mechanism needs comprehensive exploration. Network pharmacology, through screening potential oncogenic pathways and targets between drug and tumor, is widely used to theoretically explore traditional medicine’s synergistic effects on lung cancer (317–319). Furthermore, further exploration of traditional medicine may need more understanding of signaling pathways and more *in vivo* and clinical experiments. Natural products widely exist in nature, a treasure house for searching for new tumor therapeutic drugs. They deserve our attention and need further study to enhance their therapeutic effect on lung cancer.

## Author contributions

YZ: Writing – original draft. FW: Investigation, Writing – original draft. GL: Writing – review & editing. JX: Investigation, Writing – original draft. JZ: Investigation, Writing – original draft. EG: Writing – review & editing. JY: Writing – review & editing. JW: Conceptualization, Writing – original draft.

## Glossary

**Table:**

NSCLC	Non-small Cell Lung Cancer
SCLC	Small-Cell Lung Cancer
PD-1	Programmed cell death 1
PD-L1	Programmed death-ligand 1
IDO	Indoleamine-2,3-dioxygenase
DNMT1	DNA methyltransferase-1
HDAC3	Histone deacetylase 3
FRA1	FOS-related antigen 1
MHC-I	major histocompatibility complex class-I
MET	mesenchymal-epithelial transition factor
YAP	Yes-associated protein
TAZ	WW domain-containing transcription regulator 1
PKA	protein kinase A
LATS	large tumor suppressor
ADORA1	adenosine A1 receptor
ATF3	cAMP-dependent transcription factor 3
IRF-1	interferon regulatory factor-1
TET1	ten-eleven translocation methylcytosine dioxygenase 1
AHR	aromatic hydrocarbon receptor
COX-2	cyclooxygenase-2
PGE2	prostaglandin E2
LLC	Lewis lung carcinoma
TGF-β1	Transforming growth factor-β1
EMT	epithelial-mesenchymal transition
EZH2	Enhancer of Zeste homolog2
COP1	E3 ligase constitutive photomorphogenesis protein 1
KRAS	Kirsten rat sarcoma viral oncogene homologue
EML4-ALK	Echinoderm microtubule-associated protein like-4-anaplastic lymphoma kinase
EGFR	Epidermal growth factor receptor
PTEN	Phosphatase and tensin homolog
MAPK	Mitogen-activated protein kinase
IFN-γ	Interferon-γ
IFN-β	Interferon-β
HIF-1α	Hypoxia-inducible factor-1α
TGF-β	Transforming growth factor-β
IL-27	Interleukin-27
MUC1	Mucin 1
3’ UTR	3’ untranslated region
miRNAs	microRNAs
lncRNAs	Long non-coding RNAs
circRNA	Circular RNA
ZEB1	zinc-finger E-box-binding homeobox 1
TTP	AU-rich element-binding protein tristetraprolin
Ang II	Angiotensin II
HuR	Human antigen R
UROD	Uroporphyrinogen Decarboxylase
HRI	Heme-Regulated Inhibitor
ISR	integrated stress response
uORFs	upstream Open Reading Frames
eEF2K	Eukaryotic Elongation Factor 2 Kinase
uORFs	upstream Open Reading Frames
USP22	Ubiquitin-specific protease 22
USP8	Ubiquitin-specific protease 8
MARCH8	Membrane-associated RING-CH 8
CMTM6	CKLF-like MARVEL transmembrane domain containing 6
TRAF6	Tumor necrosis factor receptor-associated factor 6
GSK3α	Glycogen synthase kinase 3α
AMPK	Adenosine 5’-monophosphate-activated protein kinase
EGCG	(-)-epigallocatechin gallate
LCA	Licochalcone A
PD	Platycodin D
SAL	Sophora alopecuroides Linn
Sop	Sophocarpine
NLE	Nagilactone E
Z-GS	Z-guggulsterone
PG	Platycodon grandiflorum
NLR	neutrophil to lymphocyte ratio
PG2	Astragalus Polysaccharide Injection
PBMC	peripheral blood mononuclear cells
HYR-2	Huoxue Yiqi recipe-2
