# Supplementary material for: From immune checkpoints to therapies: understanding immune checkpoint regulation and the influence of natural products and traditional medicine on immune checkpoint and immunotherapy in lung cancer
Source: Front Immunol. 2024 Feb 15;15:1340307. doi: 10.3389/fimmu.2024.1340307 (PMC10902058; doi:10.3389/fimmu.2024.1340307)
Supplement: Supplementary file 1 [file Table_1.docx]

Supplementary Material

From Immune Checkpoints to Therapies: Understanding Immune Checkpoint Regulation and the influence of Natural Products and Traditional Medicine on Immune Checkpoint and Immunotherapy in Lung Cancer

Yibin Zhou^1^, Fenglan Wang^1^, Guangda Li^1^, Jing Xu^1^, Jingjing Zhang^1^, Elizabeth Gullen^2^, Jie Yang^1^, and Jing Wang^1^*

*** Correspondence:** Jing Wang: [jwang2936@126.com](mailto:jwang2936@126.com)

## Supplementary Tables

Supplementary Table 1 Formula of Traditional medicines.

| Studied Materials | Formula | Reference |
| --- | --- | --- |
| Bu Fei Decoction | Codonopsis pilosula, Schisandra chinensis,  Rehmannia glutinosa, Astragalus, Aster and Cortex Mori, at a  ratio of 3:2:1.8:4:2:2 | (299) |
| HYR-2 | Salvia, Panax ginseng, Liquorice | (300) |
| Qingfei Jiedu decoction | Scutellariae Barbatae Herba, Lobeliae Chinensis  Herba, Hedyotis Diffusae Herba  , Herba Solani Lyrati  , Solanum Nigrum, and Coicis Semen | (301) |
| Qiyusanlong decoction | Astragali Radix, Polygonati Odorati Rhizoma, scolopendra, pheretima, Solanum nigrum L., Hedyotis diffusa Willd, coicis semen, Euphorbia helioscopia L., Curcumae Rhizoma, Fritillariae Cirrhosae Bulbus | (302) |
| Feiji Recipe | Astragali Radix, Glehniae Radix, asparagi radix, Ophiopogonis Radix, Poria, Ligustri Lucidi Fructus, Selaginella doederleinii Hieron., Coicis Semen, Salvia chinensis Benth., Epimedii Folium, Trichosanthis Pericarpium, Paridis Rhizoma, Raunuculi Ternati Radix, Pinelliae Rhizoma, Iphigenia indica Kunth, Arisaematis Rhizoma Preparatum, Herba Houttuyniae, Prunellae Spica | (303, 304) |
| Yu-Ping-Feng | Astragali Radix, Atractylodis Macrocephalae Rhizoma, and Saposhnikoviae Radix in a weight ratio of 2:2:1 | (305) |
| Lung Cancer Fang No. 1 | Codonopsis pilosula, Radix angelicae  dahurica, Atractylodes macrocephala | (306) |
| Jianpichuji Fang | Astragalus membranaceus 30g, Codonopsis pilosula 10g, Poria 15g, Fritillariae Thunbergii Bulbus 15g, Atractylodes Macrocephala 15g, Pinellia ternate 10g, Citri Reticulatae Pericarpium 10g, Aucklandiae Radix 5g, Amomi Fructus 10g, Lilii Bulbus 10g, Platycodonis Radix 12g, Radix Padoniae Rubra 10g, Sparganii Rhizoma 5g, Curcumae Rhizoma 6g, Glycyrrhizae Radix Et Rhizoma Praeparata Cum Melle 10g | (307) |
| Yanghe Decoction | Rehmanniae Radix Praeparata 30g, Cinnamomi Cortex 5g, Ephedrae Herba 6g, Colla Cornus Cervi 12g, Sinapis Albae Semen 9g, Rhizoma Zingiberis Preparata 12g, Radix Glycyrrhizae 6g | (308) |
| Warming Spleen and Kidney Fang | Aconitum carmichaeli Debx 10g, Zingiberis Rhizoma10g, Codonopsis Radix 10g, Atractylodes Macrocephala 10g, Poria 15g, Myristicae Semen 15g, Euryales Semen 10g, Euodiae Fructus 5g, Radix Glycyrrhizae 10g, Jujubae Fructus 10g | (309) |

Abbreviation: LLC= Lewis lung carcinoma; g= grams.
